# Assessment of cancer pain in a patient with communication difficulties: a case report

**DOI:** 10.1186/s13256-016-0935-2

**Published:** 2016-06-02

**Authors:** Seiji Okimasa, Yasufumi Saito, Hiroshi Okuda, Toshikatsu Fukuda, Masatsugu Yano, Yuzo Okamoto, Eiji Ono, Hideki Ohdan

**Affiliations:** 1 Department of Surgery, Hiroshima General Hospital of West Japan Railway Company, 3-1-36, Futabanosato, Higashi-ward, Hiroshima 732-0057 Japan; 2 Department of Gastroenterological and Transplant Surgery, Applied Life Sciences, Institute of Biomedical & Health Sciences, Hiroshima University, 1-2-3, Kasumi, Minami-ward, Hiroshima 734-0037 Japan

**Keywords:** Cancer pain, Palliative care, Dementia, Cognitive impairment, Pain scale, Japan, Case report

## Abstract

**Background:**

The number of patients who have difficulty with mutual understanding has been increasing recently due to an aging society. This emerging issue needs to be addressed. We report an instructive case of a patient who had communication difficulties due to dementia and sequelae of alcoholic encephalopathy.

**Case presentation:**

A 66-year-old man of Mongolian race presented with coronary arteriosclerosis, spinal canal stenosis, transverse colon cancer, and alcoholic encephalopathy. We had been requested to remove wires that had been used for the closure of his chest in a coronary artery bypass grafting procedure. However, on admission, a tortured expression and abdominal distention were observed, along with emaciation. We diagnosed terminal stage cancer, and palliative care was offered. An abdominal computed tomographic scan revealed rectal cancer with stenosis and invasion to the adjacent tissues. A metallic stent was inserted, leading to reduction of the abdominal distention and an improvement of tachycardia. However, the patient’s tortured expression was not completely relieved; therefore, an assessment of cancer pain was considered. The Abbey Pain Scale was applied. On the basis of the patient’s score, analgesics and an opioid, among other medications, were administered. These led to relief of the patient’s tortured expression and reduced his Abbey Pain Scale score. Following this, the patient’s vital signs continued to be stable, and he was transferred to the referral institution.

**Conclusions:**

Management of cancer pain in elderly patients with mutual understanding difficulties must be performed carefully. In the case of our patient, staff at the referral institution informed us of the patient’s latent torture, and we applied the Abbey Pain Scale. There was some confusion and uncertainty regarding clinical management throughout the patient’s care; however, his condition eventually stabilized. We believe the application of the Abbey Pain Scale assists in the relief of cancer pain. However, accumulation of further cases and experiences to verify this assessment is required.

## Background

An aging society is a serious issue in Japan. In 1989, 11.6 % of the population was 65 years of age or older. In 2011, 23.1 % of the population was aged 65 years or older [1]. This change occurred in a shorter span of time than in any other country [2]. The number of people who are unable to communicate effectively due to conditions such as dementia and sequelae of brain damage has been increasing [3]. Discreet clinical management of this group of patients is required. We report a case of a 66-year-old man with rectal cancer accompanying communication difficulties due to dementia and sequelae of alcoholic encephalopathy.

## Case presentation

**Fig. 1:**
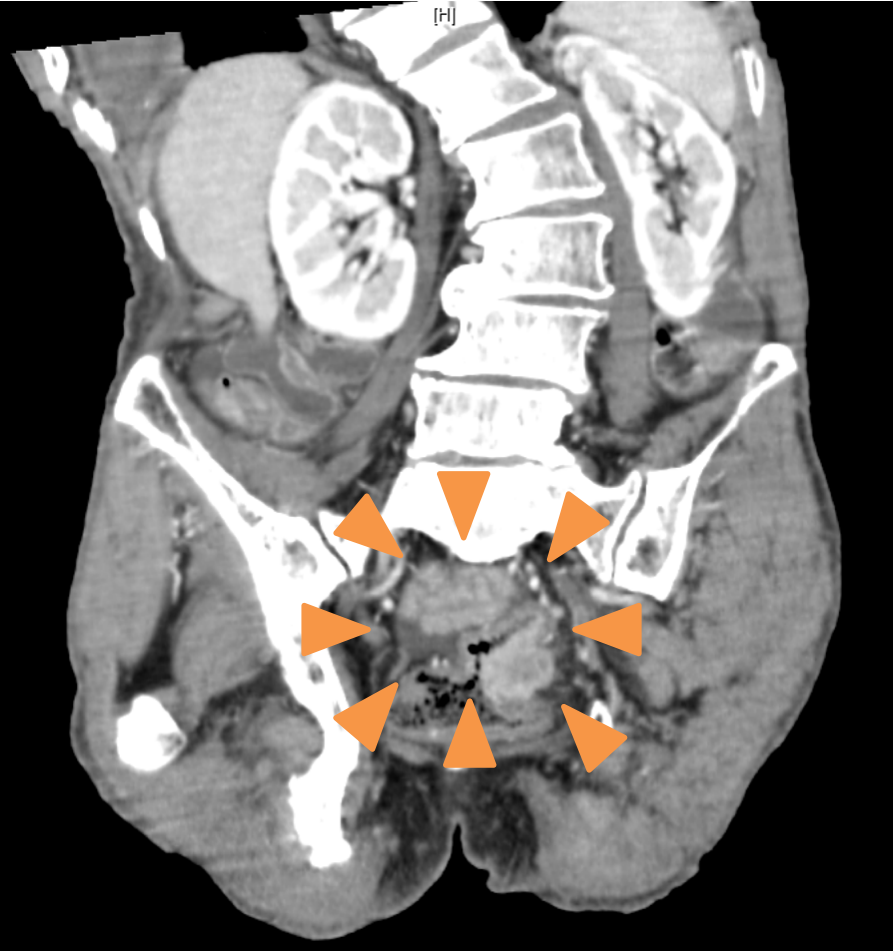
An abdominal computed tomographic scan revealing a mass lesion in the rectum (*orange arrowheads*)

**Fig. 2:**
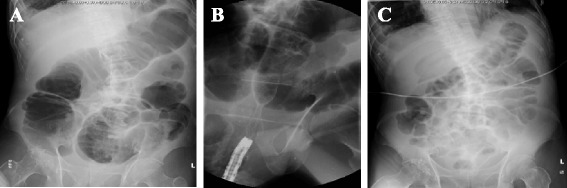
An abdominal x-ray. **a** Before decompression. **b** Placement of a metallic stent. **c** Two days after placement of a metallic stent

Our patient was a 66-year-old man of Mongolian race who had previously exhibited coronary arteriosclerosis, spinal canal stenosis, transverse colon cancer, and alcoholic encephalopathy. He was presented to our institute for the removal of wires used for the closure of his chest during a coronary artery bypass graft. However, on admission, severe abdominal distention and emaciation were observed. Emaciation caused the wires to be conspicuous along the incision line. Blood tests revealed an inflammatory response, malnutrition, anemia, and coagulation disorder. Staff at the referral institute informed us that the patient had recently been exhibiting a tortured expression. An abdominal computed tomographic scan revealed rectal cancer with stenosis and invasion to the adjacent tissues (Fig. 1). At the same time, multiple distant metastases in the lung, liver, and bone were detected. Palliative care was therefore offered. Based on an interview with the patient’s family, informed consent for best supportive care (BSC) was obtained. In regard to palliative support, placement of a metallic stent at the stenotic portion in the rectum was performed (Fig. 2). The patient’s abdominal distention was relieved by this procedure. The patient’s waist size decreased from 100 cm to 80 cm. Although this led to improvement of tachycardia, latent torture was still suspected. Staff at the referral institute informed us that his expression was not as calm as observed previously. Therefore, we felt the need to assess cancer pain. We applied the Abbey Pain Scale, a useful tool for the assessment of pain in patients with communication difficulties. In the present case, the patient’s pain score was 5, indicating that his pain was mild. Initially, acetaminophen (200 mg three times per day) and an internal liquid of morphine (5 mg twice per day) were tentatively administered. This not only calmed the patient’s voice tone and wrinkles between his eyebrows but also stabilized his heart rate. Following this, his medication was switched to fentanyl patches, a barbiturate suppository, and a Voltaren suppository (Novartis, Basel, Switzerland), among other medications, to prevent misswallowing. Gradually, the patient’s Abbey Pain Scale score, in addition to his vital signs, showed stabilization. Fortunately, rescue agents were not required. After 2 weeks of medication, the patient was returned to the referral institute. He died 1 month later, following calm days.

## Discussion

Palliative care has been gradually become recognized and accepted in Japan. The majority of patients receive palliative care in hospitals; this appears to be as favorable as medical and social support systems. However, the emerging issue of an aging society in Japan has been highlighted. An aging society is accompanied by numerous issues, such as dementia, sequelae of stroke, deterioration of athletic ability, and living alone [2]. In this report, we focus on an issue associated with an aging society: the assessment of cancer pain in a patient with communication difficulties. We report on a critical clinical issue that presents significant challenges for patient treatment and management.

In this case, the patient was a 66-year-old man of Mongolian race with plural anamneses. Alcoholic encephalopathy was a cause of his communication difficulties, alongside dementia-aggravated symptoms. His suffering was evident when he was transferred to our institute. A detailed examination revealed an obstructed ileus caused by rectal cancer accompanying distant metastases. We had an opportunity to discuss his treatment and care with his family. Informed consent for BSC was obtained because he was unable to manage a social life and receive standard chemotherapy in this condition. Placement of a metallic stent was applied for palliative care. This was effective but not completely adequate, and his tortured expression remained. Considering the latent cancer pain, we felt it necessary to assess his level of cancer pain.

We referred to clinical guidelines for cancer pain management [4] at this time. This guideline contains content on the assessment of pain for patients who are unable to communicate effectively. The Abbey Pain Scale [5], the Checklist of Nonverbal Pain Indicators (CNPI) [6], the Non-Communicative Patient’s Pain Assessment Instrument (NOPPAIN) [7], and DOLOPLUS 2 [8] are available for the assessment of pain; however, these have not yet been officially approved in Japan. The following assessments are recommended when using these methods: (1) facial expression, (2) voice and vocalization, (3) movement of the body, (4) behavior change, (5) pattern of daily life, and (6) change in mental condition. We evaluated the characteristics with the methods described above by referring to the medical literature.

The CNPI comprises six items that evaluate pain, including nonverbal vocal complaints, facial grimaces and/or winces, bracing (*e.g.*, clutching or holding onto furniture), restlessness (*e.g.*, constant or intermittent shifting of position), rubbing (*e.g.*, massaging affected area), and verbal vocal complaints (*e.g.*, ouch). A 0 is scored if the behavior is not observed, and a 1 is scored if the behavior occurs, even if briefly, during activity or at rest. However, there are no clear cutoff scores to indicate severity of pain [6].

The DOLOPLUS 2 was first described in French and is now accepted globally. This pain assessment scale comprises ten items that are divided into three subgroups (somatic, psychomotor, and psychosocial items). Each item has four response options and is scored between 0 and 3 according to the level of pain-related behavior. The overall score ranges between 0 and 30. According to this scale, pain is present when the score is equal to or greater than 5. However, this method is limited by the availability of several endpoints [8].

NOPPAIN is a general pain index developed for care staff. It is accepted as an easy-to-use tool in the assessment of pain in hospitalized, noncommunicative patients [7]. Previous studies have supported the reliability and validity of the NOPPAIN measurement tool. They suggest that this easy-to-use tool may be adequate for measuring pain indicators in older patients [9]. Although NOPPAIN is a high-quality scale, it has more checklist items to complete during the evaluation than the Abbey Pain Scale.

**Fig. 3:**
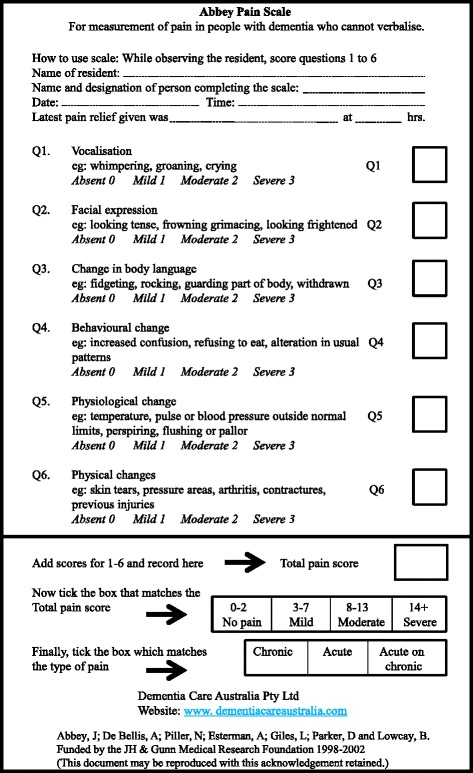
Abbey Pain Scale format. We translated the Abbey Pain Scale into Japanese and used it with our patient

There are relatively few evaluation items associated with the Abbey Pain Scale [Fig. 3], which can be applied to critical dementia patients [10, 11]. One study assessed qualitative evidence collected from users of the scale, and the findings indicated that the Abbey Pain Scale is a useful clinical device that can be completed within 1 minute [5].

## Conclusions

We believe that an easy and quick pain assessment procedure should be selected, because time should not be wasted in a critical clinical situation. Patient information should be obtained simply in order to respond as quickly as possible. Therefore, in our patient, we adopted the Abbey Pain Scale. As shown in Fig. 3, this scale comprises six items (scored from 0 to 3). According to the total score, pain severity is assessed in a range from no pain to severe pain. Although our staff showed initial concern for our patient, his vital signs stabilized with improvement of the Abbey Pain Scale score. We believe that use of this pain assessment procedure supported the stabilization of our patient.

However, there currently are several scales reported globally, and a gold standard is lacking. There is occasional uncertainty with respect to estimating breakthrough pain among noncommunicative patients, and it is this aspect that we sought to focus on. A scale needs to achieve objective accuracy on this issue. The accumulation of further experiences and information is necessary to establish a valid and appropriate scale.

## Abbreviations

BSC: best supportive care; CNPI: Checklist of Nonverbal Pain Indicators; NOPPAIN: Non-Communicative Patient’s Pain Assessment Instrument.
